# Determinants of health care utilization by immigrants in Portugal

**DOI:** 10.1186/1472-6963-8-207

**Published:** 2008-10-07

**Authors:** Sónia F Dias, Milton Severo, Henrique Barros

**Affiliations:** 1 Public Health Department, Institute of Hygiene and Tropical Medicine, New University of Lisbon, Lisbon, Portugal; 2 Department of Hygiene and Epidemiology, University of Porto Medical School, Porto, Portugal; 3 Department of Hygiene and Epidemiology, University of Porto Medical School, Porto, Portugal

## Abstract

**Background:**

The increasing diversity of population in European Countries poses new challenges to national health systems. There is a lack of data on accessibility and use of health care services by migrants, appropriateness of the care provided, client satisfaction and problems experienced when confronting the health care system. This limits knowledge about the multiple determinants of the utilization of health services. The aim of this study was to describe the access of migrants to health care and its determinants in Portugal.

**Methods:**

The study sample included 1513 immigrants (53% men), interviewed at the National Immigrant Support Centre, in Lisbon. Data were collected using questionnaires. The magnitude of associations between use of National Health Service and socio-demographic variables was estimated by means of odds ratios (OR) at 95% confidence intervals, calculated using logistic regression.

**Results:**

Among participants, 3.6% stated not knowing where to go if facing a health problem. Approximately 20% of the respondents reported that they had never used the National Health Service, men more than women. Among National Health Service users, 35.6% attended Health Centres, 12% used Hospital services, and 54.4% used both. Among the participants that ever used the health services, 22.4% reported to be unsatisfied or very unsatisfied. After adjusting for all variables, utilization of health services, among immigrant men, remained significantly associated with length of stay, legal status, and country of origin. Among immigrant women, the use of health services was significantly associated with length of stay and country of origin.

**Conclusion:**

There is a clear need to better understand how to ensure access to health care services and to deliver appropriate care to immigrants, and that special consideration must be given to recent and undocumented migrants. To increase health services use, and the uptake of prevention programs, barriers must be identified and approaches to remove them developed, through coherent and comprehensive strategies.

## Background

The greater diversity of population resulting from migratory flows poses new challenges to national health care systems [1,2]. Portugal is no exception; traditionally a country of emigration, it is now a country of immigration. The number of registered residents who do not have Portuguese citizenship has reached 6.3% of the total population, and 9% of the active population, in 2006 [3]. An undetermined number of undocumented persons are excluded in the official statistics. According to Foreigners and Borders Service of Portugal, in 2006, approximately 55% of the immigrants were male and 45% were female.

The number of immigrants is also growing almost everywhere in Europe [4,5]. This has made the use of health care services by immigrants a major international political and public health issue [6-8], particularly as regards access to health services [9-13].

The Portuguese Constitution establishes that all citizens – including immigrants – have the right to be attended in National Health Service (NHS). In principle, health care should be available to every person according to need, irrespective of nationality, economic means, legal status, or other criterion.

According to current legislation, persons without a NHS card (those without a residency or work visa) or who do not pay social security must pay the full fare for services. However, there are some exceptions; in case of a public health threat; children under 12 years of age, pregnant women and recent mothers, women in family planning programs, unemployed registered at a job centre and their dependants, recipients of welfare benefits and individuals with legally recognized chronic diseases.

The systematization and adoption of an immigration policy is recent in Portugal. The High Commission for Immigration and Intercultural Dialogue (ACIDI, I.P.) is a Public Institute with the main mission to support the integration of immigrants through inter-ministerial strategies. ACIDI has created, in 2004, National and Local Immigrant Support Centres, to propose integrated interventions, in collaboration with representative immigrant associations, social partners, and various public administration agencies and services. For instance, the Health Support Office offers the services of a team of socio-cultural mediators to improve access to health services. However, it is still unclear if and how immigrants, especially when undocumented, receive health care [12,14-16].

Even in countries where access to health care is guaranteed, immigrants do not regularly take advantage of services available. A growing body of literature indicates that immigrants face individual, socio-cultural, economic, administrative, and political barriers when using health services [13,17,18].

There is a lack of representative and comparable data on accessibility and use of health care services by immigrants, quality of care provided, client satisfaction and problems experienced when interacting with health services. This limits knowledge of the determinants of the utilization of health services [11,19], which needs to be further explored to inform planners and providers of services and to ensure equitable access to appropriate health care [1,9,20,21].

This paper presents the results of study which describes the access of migrants to health care in Portugal and its determinants.

## Methods

The study sample included 1513 immigrants (53% men) who were interviewed at the National Immigrant Support Centre (NISC), in Lisbon. Over a 1 month period, all patients attending to NISC were invited to answer the questionnaire; refusals amounted to 14%. NISC addresses integration problems faced by immigrants, regardless of legal status. It provides the services socio-cultural mediators who manage each case on the basis of particular needs, within a friendly environment. Data were collected by trained interviewers, using anonymous questionnaires, available in Portuguese and English, after an oral informed consent was obtained.

The questionnaire included items on nationality, country of origin, date of birth, length of stay in Portugal, employment**s**tatus, immigration status, educational level, housing conditions, and economic situation. There were also questions on access and utilisation of health services, and on satisfaction and perceived barriers. Approval for the study was obtained from the Ethical Committee of the University Hospital S. João.

### Data analysis

For analysis, new variables were created by aggregating categories grouping professions, perceived economic situation or country of origin. Continuous variables are presented as mean ± standard deviation. Proportions were compared using the Chi-Square and Fisher tests, as appropriate. The magnitude of the univariate and multivariate associations between use of National Health Services and socio-demographic exposures was estimated by means of odds ratios (OR) and 95% confidence intervals, calculated using logistic regression. Models were fitted separately for men and women, as gender differences in utilization were observed. To evaluate the significance of the statistical contribute of each variable for the final model the Wald test was used, and a p < 0.10 value was set to retain variables in the model. The software SPSS 14.0 was used for statistical analysis of data.

## Results

### Socio-demographic Characteristics (Table 1)

**Table 1:** Socio-demographic characteristics

	n	%
Sex		

Women	705	46.6
Men	808	53.4

Years of school education		

0–4	101	6.7
5–9	432	28.6
10–12	686	45.3
13 or more	294	19.4

Employment Status		

Unemployed	200	13.2
Student	95	6.3
Housekeeper	4	0.3
Retired	2	0.1
Employed	1212	80.1

Economic situation (perceived)		

Very insufficient	119	7.9
Insufficient	634	41.9
Sufficient	756	50.0
More than sufficient	4	0.2

Country of Origin		

Africa	522	34.8
Asia	44	2.9
Eastern Europe	178	11.9
South America	758	50.5

Legal status		

Legal	783	53.6
In process of regularization/Undocumented	678	45.4

	Mean	SD

Age (years)	33.05	8.91

Length of say in Portugal	6.55	5.76

The mean age of respondents was 33.0 ± 8.9 years. Participants in the study were predominantly from South America (50.5%, mostly from Brazil, 99.3%). The other most common origin was Africa (34.8%, mainly Cape Verde, Angola and Guinea Bissau). Around 12% of participants were from Eastern Europe (mostly from Ukraine, Romania and Moldavia), and a minority was from Asian countries (2.9%, mainly India and Pakistan).

The average length of stay in Portugal was 6.6 ± .8 years. As regards educational level, 64.7% had more than 10 years of schooling. In the sample, 80% were employed, and 50% classified their economic situation as acceptable.

### Access and utilization of health services (Table 2)

**Table 2:** Utilization and access to health services

	Total		Female		Male		
	n	%	n	%	N	%	P

Use of Health services in case of need							

Don't know	54	3.6	23	3.3	31	3.8	0.451
Health Centre	933	61.7	452	61.1	481	59.5	
Hospital	313	20.7	131	18.6	182	22.5	
Health Centre & Hospital	113	7.5	54	7.7	59	7.3	
Private medicine	79	5.2	35	5.0	44	5.4	
Other Combinations	21	1.4	10	1.4	11	1.4	

Utilization of the NHS							

Yes	1188	78.6	581	82.5	607	75.1	0.001
No	324	21.4	123	17.5	201	24.9	

Type of NHS were used*							

Health Centre	422	35.6	185	31.9	237	39.1	<0,001
Hospital	142	12.0	53	9.1	89	14.7	
Health Centre & Hospital	622	54.4	342	59.0	280	46.2	

NHS – Satisfaction level*							

Very unsatisfied	65	5.5	38	6.5	27	4.5	0.230
Unsatisfied	200	16.9	106	18.2	94	15.6	
Indifferent	99	8.4	46	7.9	53	8.8	
Satisfied	766	64.6	361	62.1	405	67.1	
Very Satisfied	55	4.6	30	5.2	25	4.1	

Barriers in access and utilization of NHS *							

None	478	40.2	209	36.0	269	44.3	0.004
Cost	40	3.4	25	4.3	15	2.5	0.107
Language	16	1.3	6	1.0	10	1.6	0.453
Distance	26	2.2	14	2.4	12	2.0	0.693
Waiting time	596	50.2	312	53.7	284	46.8	0.017
Health care providers	213	17.9	127	21.9	86	14.2	0.001
Fear of losing job	11	0.9	7	1.2	4	0.7	0.376
Other	114	9.6	65	11.2	49	8.1	0.076

*Including only the individuals that had use NHS

Among participants, 3.6% of respondents stated not to know where to go to get health services in case of need; 61.7% said they would use a Health Centre, 20.7% a Hospital, 7.5% mentioned both services. A private service was the option of 5.2%.

Approximately one fifth of the respondents reported that they had never used the National Health Service (NHS), men more frequently than women (24.9% versus 17.5%, p = 0.001). Among NHS users, 35.6% attended Health Centres, 12% used Hospital services, and 54.4% used both. NHS use was gender specific, with women reporting to use both Health Centre and Hospital more frequently (59.0% vs. 46.2%, p < 0.001), and men reporting higher use of only one type of service (Health Centre 39.1% vs. 31.9%, and Hospital 14.7% vs. 9.1%, respectively for men and women) rather than both. Among users of health services, 22.4% reported to be unsatisfied or very unsatisfied. No gender differences were observed (p = 0.230). Analysis by country of origin showed that immigrants from Eastern European countries reported to be more unsatisfied or very unsatisfied (30.3%) than individuals from Africa (20.1%) and South America (21.4%) (p = 0.001).

Barriers to appropriate and timely access were identified as waiting times (50.2%), providers' attitudes (17.9%), cost (3.4%), distance and transportation (2.2%), and language (1.3%). Women were more numerous in identifying barriers (64% vs. 55.7%, p = 0.004), mainly complaining of waiting times (53.7% vs. 46.8%, p = 0.017) and providers' attitudes (21.9% vs. 14.2%, p = 0.001). As gender differences were observed regards to these barriers, we examined the effect of country of origin stratified by gender. Providers' attitudes were recognized as barriers regardless country of origin (p = 0.152 and p = 0.863, respectively for women and men). Waiting times was mentioned more often by men from African countries (52.2%, p = 0.020) than from Eastern European and South American (35.4% and 43.0%, respectively); and women from African countries and Eastern European (58.3% and 60.9%, p = 0.025, respectively) complained more about waiting times than South American (47.6%, p = 0.025).

Considering the other barriers, respondents from Eastern European countries more frequently identified language as an obstacle to access, than those from Africa and South America (10.7% vs. 1% and 0.4%, p < 0.001). Cost, distance and transportation, or fear of losing their job were recognized as barriers regardless country of origin.

### Factors associated with the utilization of the NHS

The logistic regression analysis allowed the identification of age, length of stay, legal status and economic situation as positively associated with the use of health services (Table 3). More than 10 years of school education or being born in Eastern European or South American countries were significantly associated with a lower probability of using health services, both for males and females.

**Table 3:** Crude OR, adjusted OR for use NHS and several socio-demographic variables

	Men		Women	
	OR Crude	OR adjusted	OR Crude	OR Adjusted
	(IC95%)	(IC95%)	(IC95%)	(IC95%)

Age (years)	1.05 (1.03–1.07)	1.00 (0.98–1.03)	1.02 (1.00–1.04)	1.00 (0.98–1.03)

Length of stay (years)	1.48 (1.36–1.59)	1.43 (1.30–1.58)	1.60 (1.43–1.78)	1.58 (1.38–1.81)

Years of school education				

0–4	1	1	1	1
5–9	0.70 (0.28–1.76)	0.86 (0.30–2.48)	0.41 (0.12–1.42)	1.01 (0.24–4.24)
10–12	0.33 (0.14–0.80)	0.58 (0.21–1.64)	0.28 (0.82–0.91)	1.15 (0.28–4.66)
13 or more	0.35 (0.14–0.90)	0.60 (0.20–1.78)	0.23 (0.07–0.78)	0.93 (0.22–3.91)

Employment Status*				

Not Employed	1	--	1	--
Employed	0.85 (0.53–1.34)	--	0.96 (0.60–1.52)	--

Economic situation (perceived)				

Very insufficient/Insufficient	1.59 (1.13–2.23)	0.99 (0.65–1.51)	1.84 (1.24–2.74)	1.58 (0.50–1.26)
Sufficient/More than sufficient	1	1	1	1

Origin Country				

Africa	1	1	1	1
Eastern Europe	0.21 (0.12–0.38)	0.48 (0.25–0.94)	0.17 (0.07–0.40)	0.25 (0.10–0.67)
South America	0.23 (0.14–0.36)	0.76 (0.43–1.34)	0.12 (0.06–0.24)	0.33 (0.15–0.73)

Legal status				

Legal	4.05 (2.82–5.79)	1.71 (1.11–2.63)	3.96 (2.51–6.24)	1.38 (0.81–2.36)
Undocumented or in process of regularization	1	1	1	1

*not included in the model p higher than 0.10.

After adjusting for all variables that contributed to the use of health services, for men, utilization remains significantly associated with length of stay (OR = 1.43, IC95%:1.30–1.58, per year in Portugal), legal status (OR = 1.71, IC95%:1.11–2.62, for legal compared to undocumented), and country of origin (OR = 0.48, IC95%:0.25–0.94, for Eastern European compared to African countries).

For women, the use of health services was significantly associated with length of stay (OR = 1.60, IC95%:1.43–1.78). Being born in Eastern European countries (OR = 0.25, IC95% = 0.10–0.67, compared to African countries) was significantly associated with a lower probability of using health services.

## Discussion

Understanding the issues related with migrants' health and their utilization of health services is challenging because of gaps in databases, the heterogeneity of immigrant populations, and uncertainty about how migration affects health. Although those who migrate are often healthier than residents because of the various selection processes they face [22,23], migrants are usually exposed to several health risks. The vulnerability associated with moving to an unfamiliar environment makes access to prevention and health care services a major component of the health response of host societies [10,24,25].

Access to and actual utilization of health services is the result of a complex net of determinants [6,21]. It largely depends on how a society is able to create a user-friendly environment for immigrants [26] and to overcome the socio-economic and the subtle cultural or psychological barriers that may limit people's ability to receive care [17,27].

Rates of self-reported utilization of health services in this study reflect differences across immigrants groups. Although a large proportion of respondents reported high use of health services, 21.4% reported having never used them. A small proportion showed a lack of awareness of available health services [6], which can act as a barrier to the use of health services [17,18,28].

Our results suggest that factors associated with use of health services differ between migrant groups. Consistent with the literature, undocumented migrants are more likely to report lower utilization [7,14]. Our results are consistent with a number of studies which highlight insufficient support to guarantee access to health services [29,30]. Length of stay in Portugal is an important factor in understanding differences in patterns of health services utilization. This variable is rarely taken into account when studying utilization by immigrants, which may explain why studies repeatedly found that immigrants were more likely to use emergency services than primary care services [31,32]. Our study is consistent with others that suggest that patterns of utilization evolve from "never use", more prevalent among the recently arrived, to "utilization on a regular basis" for those who have been in the country for a longer period [33]. Patterns of health services utilisation in the host country can be influenced by the country of origin because health beliefs, previous experience of illness and health care varied according different origins. A greater understanding of the influence of country of origin on utilisation is relevant for the design of services and for resource allocation. Other unmeasured factors, like health status, may also contribute to differences in use of health care services [21,34]. Indeed, health status indicators should have been included to allow comparison between subgroups of population [12].

As shown in other studies, immigrant men underutilize health services [16,19]. The major gender difference associated with use health care services is at the level of legal status. Various hypotheses can be formulated to explain this; one is that migrant women are mostly of reproductive age and therefore more likely to use services related with sexual and reproductive health, like prenatal care, maternal and child health. In Portugal, maternal and child health care has been largely promoted, calling attention to the need of a non discriminate offer to migrants.

A substantial proportion of participants reported to be satisfied or very satisfied with health services. Patient satisfaction and ease of using health care services could be important quality and access indicators and key measures for monitoring and evaluating the performance of the health care system [35]. Although we did not collect information on reasons for participants' satisfaction with health services, several studies have indicated that ethnicity, culture and country of origin frequently affect immigrants' quality and satisfaction perception of host health services [15,17,22]. The degree of satisfaction was higher among the African and South American than among the Eastern European immigrants. These findings are consistent with different degrees of health system development and access to care in country of origin. For instance, it is possible to speculate that immigrants from countries that lack health structures may perceive health services in Portugal as more efficient. In Portugal, a large group of immigrants (African and South American) come from Portuguese speaking countries, which could contribute to explain the differences related to satisfaction observed between immigrants from different countries of origin.

In the other hand, approximately one fifth of participants who used the health services reported to be unsatisfied or very unsatisfied. Dissatisfaction with health services among migrants may reflect the non-adaptation of services to migrants' lifestyles [18]. We found that immigrant women complained more about waiting times than men. One possible explanation for this finding is that immigrant women often have full-time jobs and assume cumulative responsibilities for working and care giving, so it could be difficult to take time away from work during business hours, from care of their children or household duties to access health services. This can play a critical role because it has been pointed that the process of obtaining appointments and the prolonged waiting times can hinder patients from using health services.

In our study, approximately 18% of participants identified providers' attitudes as a barrier. Since patient satisfaction is a multidimensional construct, a number of studies have stressed the need to explore ways to overcome more subtle barriers between health providers and patients from different cultural backgrounds [17,36]. Studies have also pointed out that superficial stereotypes about migrants' heath held by providers often stand in the way of providing the best quality care [37-39]. Immigrants, on the other hand, may hold different views and expectations of health and appropriate care, based on experience with the health system in their country of origin [19,40,41]. However, reasons for these findings are complex, and more research is needed in this field.

Some of these findings may be biased due to our selection procedure. Collecting data only from migrants presenting themselves at the NISC leads to an overrepresentation of more affluent and better integrated migrant groups. On the other hand, as this centre is viewed by migrants as an independent institution dedicated to solving individual integration problems, we are confident that the sampling procedure allowed for a fairly representative sample of migrant conditions. Some immigrants for whom a translated questionnaire was not available may have been excluded from the study, though most who do not speak Portuguese or English come with a friend or family member who could offer support in the completion of survey forms. As the response rate was high, we are confident that these findings reflect the situation of a large group of immigrants living in Lisbon.

## Conclusion

There is a need to understand better how to ensure access to health services and how to deliver appropriate care to immigrants, particularly to recent and undocumented migrants [16,29,33]. Appropriate care is delivered on a continuous and integrated basis, with greater attention to prevention and health promotion [10,29,30]. This study suggests further research into immigrants' perceptions of health services to better understand their needs, barriers to access and approaches to overcome them.

A large majority of respondents reported health care services utilization and high levels of satisfaction. Although Portugal has only recently become a receiving country, initiatives for full integration of immigrants and policy regarding the health of migrants are relatively developed. Our results may indicate that these efforts work. Nonetheless, coherent and comprehensive strategies targeting migrants are needed [1,20,36,40], in terms of service reorganization and availability of culturally competent providers [25,33,37,38]. As expected from prior research, greater involvement of migrants in decision-making improves patient satisfaction and outcomes [18,28]. For recent migrants, an additional challenge is to ensure that they understand the Portuguese health care system [6]. Promoting inclusion and taking into account the values and experiences of immigrants can play a critical role to ensure that migration remains a healthy and socially productive process [28,39].

## Competing interests

The authors declare that they have no competing interests.

## Authors' contributions

All the authors have contributed to the study. SD and HB conceived the study, participated in the design of the study, in analyses and interpretation of data and wrote the manuscript. MS performed data management and main statistical analysis and helped draft the manuscript. All authors read, reviewed and approved the final manuscript.

## Pre-publication history

The pre-publication history for this paper can be accessed here:


